# Association of duration of residence in the southeastern United States with chronic kidney disease may differ by race: the REasons for Geographic and Racial Differences in Stroke (REGARDS) cohort study

**DOI:** 10.1186/1476-072X-12-17

**Published:** 2013-03-21

**Authors:** Laura Plantinga, Virginia J Howard, Suzanne Judd, Paul Muntner, Rikki Tanner, Dana Rizk, Daniel T Lackland, David G Warnock, George Howard, William M McClellan

**Affiliations:** 1 Department of Epidemiology, Rollins School of Public Health, Emory University School of Medicine, Emory University, Atlanta, GA, USA; 2 Laney Graduate School, Emory University School of Medicine, Emory University, Atlanta, GA, USA; 3 Department of Epidemiology, University of Alabama at Birmingham, Birmingham, AL, USA; 4 Department of Biostatistics, School of Public Health, University of Alabama at Birmingham, Birmingham, AL, USA; 5 Division of Nephrology, School of Medicine, University of Alabama at Birmingham, Birmingham, AL, USA; 6 Department of Medicine, Medical University of South Carolina, Charleston, SC, USA; 7 Division of Nephrology, Emory University School of Medicine, Emory University, Atlanta, GA, USA

**Keywords:** Albuminuria, Kidney function, End-stage renal disease, Stroke belt, African-American, Geographic variation

## Abstract

**Background:**

Prior evidence suggests that longer duration of residence in the southeastern United States is associated with higher prevalence of diabetes and hypertension. We postulated that a similar association would exist for chronic kidney disease (CKD).

**Methods:**

In a national population-based cohort study that enrolled 30,239 men and women ≥ 45 years old (42% black/58% white; 56% residing in the Southeast) between 2003 and 2007, lifetime southeastern residence duration was calculated and categorized [none (0%), less than half (>0-< 50%), half or more (≥50-< 100%), and all (100%)]. Prevalent albuminuria (single spot urinary albumin:creatinine ratio of ≥30 mg/g) and reduced kidney function (estimated glomerular filtration rate <60 ml/min/1.73 m^2^) were defined at enrollment. Incident end-stage renal disease (ESRD) during follow-up was identified through linkage to United States Renal Data System.

**Results:**

White and black participants most often reported living their entire lives outside (35.7% and 27.0%, respectively) or inside (27.9% and 33.8%, respectively) the southeastern United States. The prevalence of neither albuminuria nor reduced kidney function was statistically significantly associated with southeastern residence duration, in either race. ESRD incidence was not statistically significantly associated with all vs. none southeastern residence duration (HR = 0.50, 95% CI, 0.22-1.14) among whites, whereas blacks with all vs. none exposure showed increased risk of ESRD (HR = 1.63, 95% CI, 1.02-2.63; *P*_*raceXduration*_ = 0.011).

**Conclusions:**

These data suggest that blacks but not whites who lived in the Southeast their entire lives were at increased risk of ESRD, but we found no clear geographic pattern for earlier-stage CKD.

## Introduction

Chronic kidney disease (CKD), defined by albuminuria or reduced estimated glomerular filtration rate (eGFR), is common among adults in the United States (estimated prevalence >10%) [1], and, in 2009, more than 600,000 individuals with CKD were treated for end-stage renal disease (ESRD) [2], requiring dialysis or kidney transplantation to sustain life. Substantial geographic variation exists in the age-, race- and sex-adjusted incidence of ESRD in the United States, and many of the southeastern states (which overlay the so-called “stroke belt” [3]) have higher incidence than many other U.S. regions [2]. Further, both diabetes [4] and hypertension [5], which are strong risk factors for CKD, have been shown to be more prevalent in the southeastern United States, compared to the rest of the country. This raises the possibility that regional factors that are associated with increased risk of developing diabetes and hypertension during one’s lifetime, as well as the diseases themselves, might contribute to a higher risk of CKD and ESRD among residents of this region. Factors that could vary by region and also affect these risks include behavioral (*e.g*., diet and physical activity), environmental (*e.g*., water quality and food availability), and possibly unknown population-level genetic factors.

Despite increasing interest in the geographic aspects of CKD epidemiology [6], there remains little known about the associations of the duration of residence in the southeastern United States with prevalence of CKD and incidence of ESRD. Additionally, since diabetes and hypertension have also been shown to be more prevalent among blacks compared to whites [7], the degree to which race may affect such associations is of interest. Thus, we hypothesized that, in a national population-based cohort of community-dwelling individuals aged ≥45 years [the REasons for Geographic and Racial Differences in Stroke (REGARDS) cohort], longer duration of residence in the Southeast would be associated with higher prevalence of albuminuria and reduced kidney function and incidence of ESRD in both blacks and whites.

## Results

### Participant characteristics

Participants with complete residence information had a mean age of 65 years; overall, approximately 56% were female, 40% were black, and 35% were college graduates. In general, females, those without a college education, currently living in a non-urban location, with diabetes, and with hypertension had higher southeastern residence duration. Participant characteristics by southeastern residence duration and race are shown in Table 1. Black participants were more likely to be younger and female and to have less education, lower income, lower residential mobility, urban residence, higher BMI, diabetes and hypertension, compared to whites; they were less likely to use NSAIDs regularly. On average, blacks had higher albumin:creatinine ratios and eGFRs than whites.

**Table 1:** Cohort participant characteristics, by race and southeastern residence duration categories

**Southeastern residence duration:**	**White**					**Black**				
	**Total**	**None**	**<Half**	**≥Half**	**All**	**Total**	**None**	**<Half**	**≥Half**	**All**
***N *(%)**	16,381	5,851 (35.7)	2,447 (14.9)	3,509 (21.4)	4,574 (27.5)	10,689	2,885 (27.0)	2,566 (24.0)	1,626 (15.2)	3,612 (33.8)
**Demographic**										
Mean age in years (SD)	65.5 (9.5)	65.9 (9.7)	65.5 (9.1)	65.4 (9.1)	65.1 (9.5)	64.1 (9.2)	64.4 (9.4)	66.1 (8.8)	62.5 (8.7)	63.0 (9.2)
% female	50.5	47.4	44.1	52.0	56.6	63.9	63.3	59.4	61.6	68.7
**Socioeconomic status**										
% college graduate	41.4	45.8	50.7	42.7	29.6	26.2	28.8	24.4	30.1	23.7
Annual income										
% < $35,000	34.4	32.3	29.3	35.0	39.6	52.8	49.4	51.7	51.8	57.0
% ≥ $35,000	53.4	55.6	59.8	53.7	47.0	34.7	38.9	36.6	37.6	28.8
% refused	12.1	12.1	11.0	11.3	13.3	12.4	11.8	11.8	10.7	14.2
**Residence**										
% 5+ lifetime moves^a^	50.2	46.9	74.9	69.7	26.2	17.6	14.7	21.4	37.6	8.2
% ≥20 years at current residence	53.8	54.0	33.7	61.1	58.6	64.9	62.9	68.7	70.3	61.5
% urban	69.3	81.8	67.1	65.9	56.4	90.4	99.0	96.0	82.4	83.1
**Access**										
% insured	95.8	96.5	95.9	95.7	94.8	90.3	92.2	93.2	89.1	87.3
% with regular medical care	84.3	84.4	83.1	84.4	84.6	81.7	80.9	81.4	82.5	82.3
**Clinical**										
% current smoker	12.3	10.9	11.8	13.8	13.2	17.2	18.3	16.9	19.4	15.6
% obese (BMI >30 kg/m^2^)	30.9	30.4	30.5	29.2	33.2	48.7	46.9	46.3	47.2	52.4
% using NSAIDS	16.3	15.6	15.2	17.8	16.7	11.9	11.7	11.2	12.2	12.3
% using ACEI/ARBs	31.6	29.0	30.8	32.8	34.4	42.0	39.4	40.6	41.1	45.3
% with diabetes	15.3	13.2	14.3	16.2	18.0	30.3	28.1	27.4	29.9	34.3
% with hypertension	50.3	47.3	49.7	51.3	53.7	71.7	69.0	72.3	69.6	74.3
% family history of ESRD	4.7	4.3	4.1	4.8	5.6	10.7	8.8	9.5	11.2	12.7
**Mean ACR**^b^**(SD)**	9.30 (2.94)	9.21 (2.92)	8.76 (2.77)	9.39 (3.06)	9.68 (3.06)	11.94 (4.06)	12.18 (4.06)	11.70 (4.06)	10.59 (3.82)	12.55 (4.18)
**Mean eGFR (SD)**	82.6 (17.1)	82.1 (17.0)	83.2 (16.4)	82.8 (17.1)	82.8 (17.5)	88.7 (23.3)	88.9 (23.5)	86.5 (22.8)	90.4 (21.8)	89.4 (24.1)

Southeastern residence duration: total, all participants; none, 0% of life spent in Southeast; <half, >0%-50% of life spent in Southeast; ≥half, ≥50%-< 100% of life spent in Southeast; all, 100% of life spent in Southeast. The Southeast included North Carolina, South Carolina, Georgia, Alabama, Mississippi, Tennessee, Arkansas, and Louisiana; other included the 40 other contiguous U.S. states (excluding Alaska and Hawaii).

BMI, body mass index (kg/m^2^); ACEI, angiotensin-converting enzyme inhibitor; ARB, angiotensin receptor blocker; NSAID, non-steroidal anti-inflammatory drug; ACR, albumin:creatinine ratio (mg/g); eGFR; estimated glomerular filtration rate (ml/min/1.73 m^2^). Diabetes and hypertension were defined by self-reported diagnoses; self-reported use of insulin or oral hypoglycemic and anti-hypertensive medications; or measured fasting blood glucose ≥126 mg/dl or random glucose ≥200 mg/dl and blood pressure >140/>90 mmHg, respectively.

Missing data (whites and blacks): education, *n* = 19; insurance; *n* = 21; routine source of medical care, *n* = 970; urbanization of current residence, *n* = 2,612; smoking, *n* = 105; BMI, *n* = 187; regular use of NSAIDs, *n* = 101; diabetes, *n* = 969; hypertension, *n* = 65; albuminuria, *n* = 1273; eGFR, *n* = 1105. For those variables with >10% of data missing, ‘unknown’ was included as a category in the table.

*P* < 0.001 in whites and blacks across southeastern exposure categories, by chi-square (categorical) and ANOVA (continuous) tests, for all characteristics shown, except routine site for medical care (*P* > 0.05 for whites and blacks).

^a^Lifetime moves comprise moves to the Southeast, to the rest of the United States, and outside of the United States, regardless of duration.

^b^Geometric mean.

**Figure 1:**
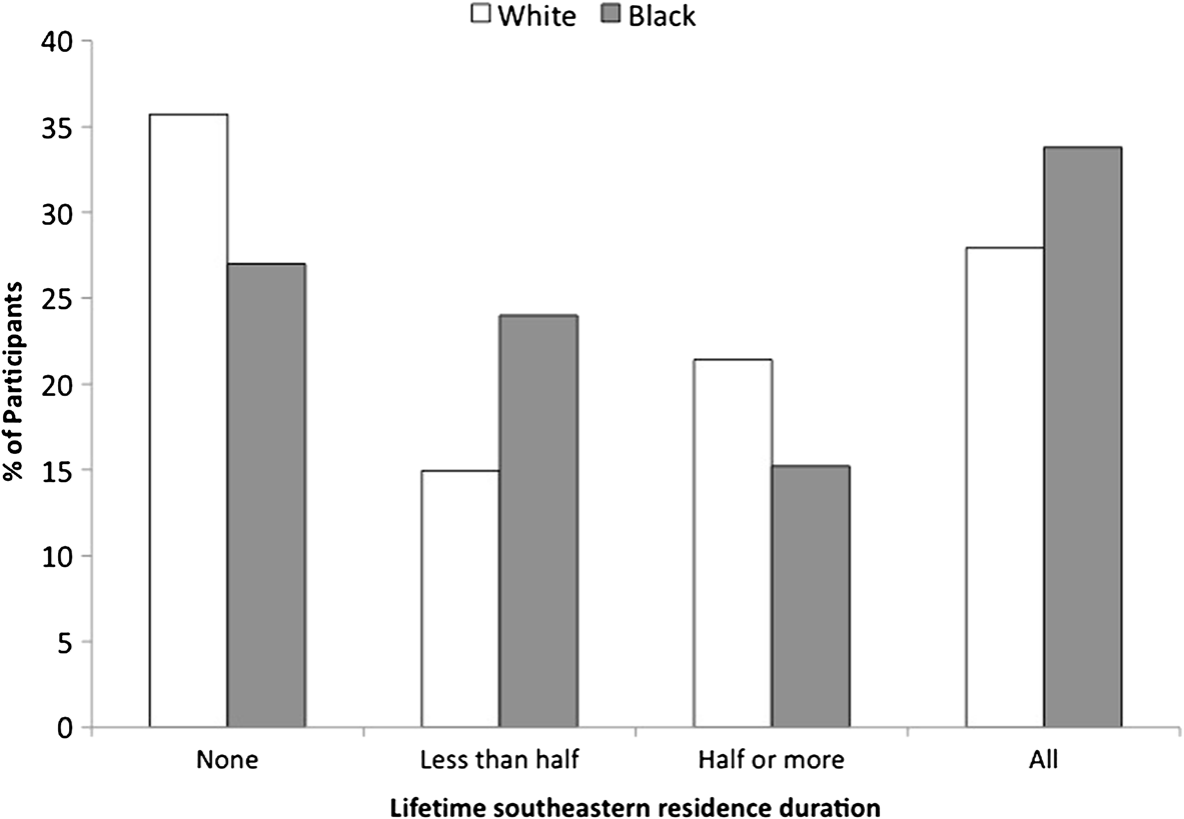
**Crude distribution of lifetime southeastern residence duration, by race. **Bars, crude percentage of participants in each category. Southeastern U.S. states included: North Carolina, South Carolina, Georgia, Alabama, Mississippi, Tennessee, Arkansas, and Louisiana; other included the 40 other contiguous U.S. states (excluding Alaska and Hawaii).

The majority of participants had spent their entire lifetime either outside (32.3%) or inside (30.3%) the Southeast. The median southeastern residence duration was 46.8% (47.8% for whites and 45.8% for blacks; 33.9% for males and 59.4% for females). Figure 1 shows that the majority of participants spent 100% of their lives (“all”) or 0% of their lives (“none”) in the Southeast. White participants more often, and black participants less often, reported no vs. entire life in the Southeast (whites, 35.7% vs. 27.9%; blacks, 27.0% vs. 33.8%).

### Prevalence of albuminuria by southeastern residence duration

Albuminuria was more prevalent in both whites and blacks who either spent none or all of their life in the Southeast, relative to those who spent part but not all of their life in the region (Table 2). Additionally, the crude prevalence of albuminuria was higher in blacks than whites in every southeastern residence duration category. The PORs, relative to the “none” (0%) southeastern residence duration category, show somewhat increased prevalence for the “all” (100%) southeastern residence duration category. For example, with adjustment for age and sex and compared to no southeastern residence, whites who spent all of their lives in the Southeast had a POR of 1.16 (95% CI, 1.02-1.31). However, this association became non-significant with further adjustment for clinical factors and SES (Table 3). In the black population, we found no pattern of higher prevalence with longer duration of southeastern residence. Rather, blacks with lifetime southeastern residence duration less than half (>0% and <50%) and half or more (≥50% and <100%) had age- and sex-adjusted PORs of 0.87 (95% CI, 0.76-1.00) and 0.80 (95% CI, 0.68-0.95), respectively, relative to blacks with no residence in the region, and these associations persisted with further adjustment. The statistical significance of the interaction by race of the association between southeastern exposure and albuminuria prevalence was attenuated with adjustment for SES.

**Table 2:** Crude prevalence of albuminuria and reduced kidney function and crude incidence of end-stage renal disease, by race and southeastern residence duration categories

**Outcome**	**Lifetime southeastern residence duration**									
	**White**					**Black**				
	**None**	**<Half**	**≥Half**	**All**	**P**	**None**	**<Half**	**≥Half**	**All**	**P**
**Crude prevalence of albuminuria**										
No. of events	670	245	399	560	0.044^a^	534	443	243	671	0.037^a^
No. of individuals	5619	2348	3377	4380	0.149^b^	2728	2431	1541	3373	0.876^b^
Prevalence (%)	11.9	10.4	11.8	12.8		19.6	18.2	15.8	19.9	
**Crude prevalence of reduced kidney function**										
No. of events	619	218	347	497	0.042^a^	316	301	152	417	0.067^a^
No. of individuals	5640	2364	3414	4418	0.670^b^	2714	2443	1552	3420	0.895^b^
Prevalence (%)	11.0	9.2	10.2	11.3		11.6	12.3	9.8	12.2	
**Crude incidence of end-stage renal disease**										
No. of events	23	5	12	8	0.278^a^	30	29	15	48	0.076^a^
No. of individuals	5839	2447	3504	4564	0.192^b^	2866	2537	1618	3575	0.009^b^
Incidence (per million person-years)	1149.5	589.8	1007.6	536.1		2829.2	3152.7	2891.9	4262.9	

Southeastern U.S. states included: North Carolina, South Carolina, Georgia, Alabama, Mississippi, Tennessee, Arkansas, and Louisiana; other included the 40 other contiguous U.S. states (excluding Alaska and Hawaii).

^a^By *χ*^2 ^(albuminuria, reduced kidney function) and log-rank (end-stage renal disease) tests of equality across southeastern exposure categories.

^b^By Cochran-Armitage (albuminuria, reduced kidney function) and log-rank (end-stage renal disease) tests for trend across southeastern exposure categories.

**Table 3:** Crude and adjusted prevalence odds ratios for albuminuria and reduced kidney function and hazard ratios for incident ESRD, by race and southeastern residence duration categories

**Model**	**Lifetime southeastern exposure**								***P***^a^
	**White**				**Black**				
	**None**	**<Half**	**≥Half**	**All**	**None**	**<Half**	**≥Half**	**All**	
Prevalence odds ratios (95% CI) for **albuminuria**									
Unadjusted	Ref.	0.86 (0.74-1.01)	0.99 (0.87-1.13)	1.08 (0.96-1.22)	Ref.	0.92 (0.80-1.05)	0.77 (0.65-0.91)	1.02 (0.90-1.16)	0.054
Adjusted^b^									
+ demographics	Ref.	0.87 (0.75-1.02)	1.04 (0.91-1.18)	1.16 (1.02-1.31)	Ref.	0.87 (0.76-1.00)	0.80 (0.68-0.95)	1.08 (0.95-1.22)	<0.001
+ SES	Ref.	0.89 (0.76-1.04)	1.02 (0.89-1.17)	1.08 (0.96-1.23)	Ref.	0.84 (0.73-0.97)	0.78 (0.66-0.92)	0.99 (0.86-1.13)	0.178
+ clinical	Ref.	0.86 (0.73-1.01)	0.94 (0.82-1.08)	1.00 (0.88-1.14)	Ref.	0.86 (0.74-1.00)	0.74 (0.62-0.89)	0.92 (0.80-1.05)	0.265
Prevalence odds ratios (95% CI) for **reduced kidney function**									
Unadjusted	Ref.	0.82 (0.70-0.97)	0.92 (0.80-1.05)	1.03 (0.91-1.17)	Ref.	1.07 (0.90-1.26)	0.82 (0.67-1.01)	1.05 (0.90-1.23)	0.051
Adjusted^b^									
+ demographics	Ref.	0.97 (0.81-1.15)	0.98 (0.80-1.22)	1.19 (1.03-1.40)	Ref.	0.89 (0.75-1.05)	1.00 (0.87-1.16)	1.13 (0.99-1.30)	0.843
+ SES	Ref.	0.94 (0.79-1.13)	0.96 (0.77-1.18)	1.11 (0.94-1.31)	Ref.	0.91 (0.76-1.08)	0.99 (0.86-1.05)	1.08 (0.94-1.23)	0.937
+ clinical	Ref.	0.96 (0.81-1.15)	0.92 (0.74-1.14)	1.06 (0.80-1.26)	Ref.	0.88 (0.74-1.35)	0.93 (0.80-1.08)	1.00 (0.87-1.15)	0.878
Hazard ratios (95% CI) for **incident end-stage renal disease**									
Unadjusted	Ref.	0.51 (0.19-1.34)	0.91 (0.45-1.84)	0.53 (0.24-1.18)	Ref.	1.16 (0.70-1.93)	1.11 (0.60-1.06)	1.73(1.10-2.74)	0.014
Adjusted^b^									
+ demographics	Ref.	0.52 (0.20-1.36)	0.94 (0.47-1.89)	0.55 (0.25-1.24)	Ref.	1.16 (0.69-1.93)	1.11 (0.60-2.07)	1.74(1.10-2.75)	0.013
+ SES	Ref.	0.54 (0.21-1.44)	0.93 (0.46-1.88)	0.51 (0.23-1.14)	Ref.	1.17 (0.70-1.96)	1.09 (0.59-2.04)	1.66 (1.04-2.66)	0.013
+ clinical	Ref.	0.51 (0.19-1.36)	0.91 (0.45-1.84)	0.50 (0.22-1.14)	Ref.	1.23 (0.74-2.06)	1.02 (0.54-1.93)	1.63 (1.02-2.61)	0.011

Southeastern U.S. states included: North Carolina, South Carolina, Georgia, Alabama, Mississippi, Tennessee, Arkansas, and Louisiana; other included the 40 other contiguous U.S. states (excluding Alaska and Hawaii).

^a^*P *for interaction by Wald *χ*^2 ^test between race and overall percentage of lifetime lived in the southeastern United States.

^b^Demographics: age, gender, race (overall models only); SES: education, income clinical; diabetes, hypertension (by self-reported medications or glucose/BP).

In sensitivity analyses, income-stratified analyses (Table 4) showed that the lack of association of albuminuria with southeastern residence duration was similarly present in high (>$35,000)- and low (<$35,000)-income groups. Neither the three-way interaction term of income, race, and southeastern residence duration nor two-way interactions of race with southeastern residence duration in stratified models were statistically significant. When residence duration was categorized in evenly spaced intervals of length 20% (excluding the 0% and 100% categories), results were similar: having a southeastern residence duration of 80-99% was associated with a fully adjusted POR of 0.71 (95% CI, 0.58-0.88), relative to 0% among blacks, but no other residence duration category was associated with prevalent albuminuria in blacks; no associations were seen among whites. Additionally, whether participants had spent the first 18 years of life in the Southeast (vs. not) was not associated with albuminuria: fully adjusted POR = 1.02 (95% CI, 0.94-1.13) and 0.94 (95% CI, 0.84-1.05), for whites and blacks, respectively. Finally, the age × duration term was not statistically significant for whites (*P* = 0.99) or blacks (*P* = 0.42) in fully adjusted models.

**Table 4:** **Adjusted**^**a**^**prevalence odds ratios for albuminuria and reduced kidney function and hazard ratios for incident ESRD, by income, race, and southeastern residence duration**

Income stratum	**Lifetime southeastern residence duration**								***P***_***raceXduration***_^b^	***P***_***incomeXraceXduration***_^c^
	**White**				**Black**					
	**None**	**<Half**	**≥Half**	**All**	**None**	**<Half**	**≥Half**	**All**		
Prevalence odds ratios (95% CI) for **albuminuria**										
Income < $35,000	Ref.	0.97 (0.74-1.26)	0.99 (0.80-1.23)	1.01 (0.83-1.26)	Ref.	0.85 (0.70-1.04)	0.75 (0.60-0.95)	0.94 (0.78-1.12)	0.415	0.538
Income ≥ $35,000	Ref.	0.86 (0.68-1.07)	0.89 (0.72-1.09)	1.02 (0.84-1.23)	Ref.	0.86 (0.66-1.11)	0.71 (0.52-0.98)	0.98 (0.76-1.26)	0.859	
Prevalence odds ratios (95% CI) for **reduced kidney function**										
Income < $35,000	Ref.	0.86 (0.65-1.14)	0.94 (0.75-1.18)	1.06 (0.87-1.29)	Ref.	0.97 (0.77-1.22)	1.00 (0.76-1.32)	1.02 (0.82-1.27)	0.838	0.053
Income ≥ $35,000	Ref.	0.79 (0.61-1.01)	0.89 (0.71-1.11)	0.82 (0.66-1.03)	Ref.	0.99 (0.71-1.39)	0.59 (0.37-0.94)	1.17 (0.83-1.64)	0.034	
Hazard ratios (95% CI) for **incident end-stage renal disease**										
Income < $35,000	Ref.	0.50 (0.11-2.28)	1.16 (0.47-2.89)	0.43 (0.14-1.38)	Ref.	1.57 (0.81-3.04)	1.51 (0.70-3.28)	1.98 (1.08-3.64)	0.069	0.028
Income ≥ $35,000	Ref.	0.66 (0.18-2.46)	0.56 (0.15-2.07)	0.79 (0.24-2.56)	Ref.	0.82 (0.32-2.10)	0.20 (0.03-1.53)	0.56(0.17-1.82)	0.797	

Southeastern U.S. states included: North Carolina, South Carolina, Georgia, Alabama, Mississippi, Tennessee, Arkansas, and Louisiana; other included the 40 other contiguous U.S. states (excluding Alaska and Hawaii).

^a^Demographics: age, gender, race (overall models only); SES: education, income clinical; diabetes, hypertension (by self-reported medications or glucose/BP).

^b^*P *for interaction by Wald *χ*^2 ^test for race × southeastern residence duration, within income category.

^c^*P *for interaction by Wald *χ*^2 ^test for income × race × southeastern residence duration. All lower-order interaction terms (income × race, income × duration, and race × duration) were also included in these models to keep them hierarchically well-formulated.

### Prevalence of reduced kidney function by southeastern residence duration

Crude prevalence of reduced kidney function was similar across all southeastern residence duration categories, regardless of race (Table 2). For most categories, blacks had higher prevalence of reduced kidney function than whites (Table 2). For example, in the 100% southeastern residence duration category, prevalence of reduced kidney function was 12.2% and 11.3% in blacks and whites. Table 3 shows that duration of southeastern residence was not associated with odds of reduced kidney function among whites. Similarly, among blacks, we found no dose-response pattern. Additionally, tests for interactions of race with southeastern residence duration were not statistically significant.

Sensitivity analyses showed a similar lack of association of southeastern residence with reduced kidney function was seen when the stricter cutoff of eGFR < 45 ml/min/1.73 m^2^ was used (data not shown). Income-stratified analyses (Table 4) showed that the associations of reduced kidney function with southeastern residence duration were similarly null in high- and low-income groups, with the exception of the more than half vs. none southeastern residence category in blacks of high income. With evenly spaced cutoffs (intervals of 20%, excluding the 0% and 100% categories), results were similarly null, compared to those reported in the main analyses (data not shown). Also, whether participants had spent the first 18 years of life in the Southeast (vs. not) was not associated with reduced kidney function: POR = 1.00 (95% CI, 0.89-1.12) and 0.96 (95% CI, 0.84-1.09), for whites and blacks, respectively. Finally, the age × duration term was not statistically significant for whites (*P* = 0.90) or blacks (*P* = 0.86).

### Incidence of ESRD by southeastern residence duration

Among whites, the crude incidence of ESRD was lowest in the all lifetime southeastern residence duration category and highest in the none category (536.1 and 1149.5 per million patient-years, respectively; Table 2). However, this difference was not statistically significant. In contrast, among blacks, those who had spent their entire life in the Southeast had the highest crude incidence of ESRD and those who had never lived in the region had the lowest incidence rate (4262.9 and 2829.2 per million patient-years, respectively; Table 2). Most categories of southeastern residence were not statistically significantly associated with ESRD incidence (Table 3). Blacks who lived their entire lives in the Southeast had increased risk of ESRD relative to those who never lived in the Southeast (fully adjusted POR vs. none, 1.63; 95% CI, 1.02-2.61). The statistical interaction of race with southeastern residence duration in this fully adjusted model was statistically significant (*P*_*interaction*_ = 0.011).

In sensitivity analyses, income-stratified analyses (Table 4) showed that the associations of ESRD with southeastern residence duration were similarly non-statistically significant in high- and low-income groups. Associations were similar in the two income groups among whites. Among blacks, HRs were suggestive of a harmful effect of greater southeastern residence duration in those with low income but a protective effect in those with high income. The three-way interaction term of income, race, and southeastern residence duration was statistically significant (*P* = 0.028). When residence duration was categorized in evenly spaced intervals of length 20% (excluding the 0% and 100% categories), results were similar: only the 100% residence category was associated with greater risk of ESRD among blacks, with a HR of 1.63 (95% CI, 1.02-2.61). The first 18 years spent in the Southeast (vs. not) was protective in whites (HR = 0.66, 95% CI, 0.35-1.26) and harmful in blacks (HR = 1.24, 95% CI, 0.86-1.79), but neither association was statistically significant. Finally, the age × duration term was statistically significant among whites (*P* = 0.02) but not blacks (*P* = 0.37). The significance of the interaction term appeared to be due to a lack of events in the <50% duration category among younger whites and not due to any overall difference between older and younger whites in the association of duration with risk of incident ESRD, as the HRs for 100% vs. 0% categories were quite similar among whites <65 and ≥65 years old: 0.50 (95% CI, 0.14,1.81) and 0.44 (95% CI, 0.14,1.35), respectively.

## Discussion

In this national cohort, we found that higher prevalence of albuminuria and reduced kidney function was not associated with longer duration of Southeastern residence among either whites or blacks. Results were suggestive of an association between lifetime southeastern residence duration and increased risk of ESRD, but only among blacks. The interaction between race and southeastern residence duration for ESRD suggests effect modification by race, such that lifetime exposure to the Southeast appeared to be harmful for blacks but not whites. This effect modification persisted among those with lower but not higher income.

Diabetes and hypertension account for the majority of ESRD in the United States [2]. Studies of diabetes have shown that it is more common among those living in the Southeast, often referred to as the “stroke belt” [3]. In fact, a “diabetes belt” [4] that overlays the stroke belt has recently been proposed, based upon national survey prevalence estimates. Hypertension has also been associated with the duration of residence in the stroke belt in a dose-response manner (*i.e*., higher lifetime exposure to the stroke belt was associated with higher prevalence of hypertension) [5]. In addition, racial disparities in both diabetes and hypertension persist, with blacks having higher prevalence of both conditions compared to whites in the United States [7]. Given these established associations of diabetes and hypertension—strong risk factors for development and progression of CKD—with the U.S. southeastern states and race, we expected that longer residence in this region would be associated with higher prevalence of CKD and incidence of ESRD, and that the associations might be even stronger in blacks compared to whites.

However, in this national population-based cohort, while we did observe increasing prevalence of diabetes and, to a lesser extent, hypertension with higher duration of residence in the Southeast, the same was not true for the prevalence of albuminuria and reduced kidney function, or for incident ESRD. Differential CKD progression may partially explain these results. Faster progression among blacks is postulated to be responsible for decreased prevalence of CKD but increased ESRD incidence reported among blacks as compared to whites [8]. Mortality also differs by race: blacks with CKD have higher mortality at every stage of CKD than comparable whites [9]. Thus, racial differences in CKD prevalence and CKD-related mortality do not seem to account for the differences in progression to ESRD [9,10]. However, higher early mortality and faster progression among blacks with CKD [11] may mean that this study, which recruited community-dwelling adults ≥45 years of age, was less likely to capture blacks with earlier vs. advanced-stage CKD. Additionally, if those with rapidly progressing CKD are more or less sensitive to geographic risk factors than those with slowly progressing CKD and were less likely to be captured in our study, our results may be under- or over-estimating the effect of southeastern residence duration on CKD, particularly among blacks.

Other important factors may partially account for our observed results. For example, higher income could offset deleterious effects of regional exposure in many ways, including: increasing quality of medical care received; facilitating higher adherence to healthier lifestyles and prescribed medical therapies; and improving residential neighborhood, in terms of crime, education, or pollution. Indeed, CKD has been shown to be more common in those with lower income, particularly among blacks [12-14]. Here, we found that the potential effect modifications by race for ESRD incidence were strongest in those with lower income. Those with lower income may have fewer opportunities to move for better educational and job opportunities, which in turn may affect their risk for incidence and progression of kidney disease.

Also, lifetime exposure (or cumulative life course exposure) to the southeastern United States may not be as important as other geographic factors, including early regional exposure (or critical period exposure). In fact, Howard *et al.*[5] reported that early stroke belt exposure (place of birth, in childhood and/or adolescence) was more strongly associated with hypertension in REGARDS, compared to other periods. The authors found that early stroke belt exposure was a strong risk factor, particularly among black participants; whereas lifetime exposure was more strongly associated with hypertension in white participants. Similarly, southeastern U.S. place of birth and adult residence were both shown to be independently associated with stroke mortality, although childhood exposure was not available in this study [15]. However, we found no association between southeastern residence during the first 18 years of life and prevalence of albuminuria or reduced kidney function. And, while protective and harmful effects were suggested for ESRD among whites and blacks, respectively, these results were neither statistically significant nor substantially different (in either race) from those obtained in our main analyses of lifetime exposure.

Residential mobility may also play a role. In general, higher residential mobility is thought to be associated with poorer health [16,17] and there is some evidence of an association between higher mobility and higher prevalence of hypertension [18]. However, it has been suggested that such an effect is highly dependent on age and health status at the time of migration [19]. Our results among blacks suggest that increased residential mobility, at least in and out of the Southeast, may be partially protective against albuminuria, although the reverse effect (declining health leading to decreased mobility) cannot be entirely ruled out since the duration of albuminuria is unknown. Whether this can be explained by the reasons for moving—*e.g*., educational or occupational opportunities—or by explanations such as the “healthy migrant” effect or “salmon bias,” which are often invoked to explain superior health status of immigrant populations [19,20], requires further research.

While these results demonstrate geographical variation specifically within the United States, they are likely to be relevant to international populations as well. For example, in Japan, another highly industrialized country, incidence rates of ESRD were shown to be higher in the northern vs. southern regions, mirroring known regional patterns of hypertension and stroke, despite a relatively racially homogenous population compared to the United States [21]. However, recent studies show that the geographic differences in ESRD incidence have all but disappeared in the last decade in Japan [22], suggesting that interventions may be effective in reducing geographic disparities. In India, the identification of such existing geographic variations, as was seen with the national Indian CKD registry data, particularly with respect to the prevalence of hypertensive CKD [23], is critical to development and implementation of interventions that could reduce or eliminate disparities, as has occurred in Japan. Population genetic studies could help identify global subgroups, such as those of sub-Saharan African ancestry, who may be at higher risk [24]. Such populations could be targeted for early interventions that could prevent the development of geographic disparities as these populations transition to having chronic disease as the main population health concern [25].

In addition to the limitations discussed above, there are other limitations worthy of mention. First, residence was self-reported and recall of all relocations to exact year may be somewhat flawed. Also, this exposure reflects duration of residence in the Southeast but not necessarily the amount of exposure to cultural, lifestyle, and/or environmental factors of the region, which may account for increased risk of disease, including CKD. Cohort effects could also an issue, although interactions between age and duration of southeastern residence were generally non-statistically significant, except in the models of ESRD in whites. Both albuminuria and reduced kidney function are based upon single measurements and some misclassification of CKD is likely. Without good measures of diabetes and hypertension control over time, we cannot ascertain whether these differ geographically and what effect these might have on CKD prevalence and ESRD incidence by region. Additionally, unmeasured genetic and environmental factors cannot be examined to determine the contributions of each to observed differences. ESRD is rare, leading to small numbers of events and possibly unstable estimates in southeastern exposure categories, particularly for income-stratified models. As with all observational studies, causal inference is limited. However, this study also has several strengths, including a large study sample size with adequate follow-up, ascertainment of ESRD through active follow-up, and a sampling scheme that provided adequate numbers of participants in both race and Southeastern residence.

## Conclusions

We found three unexpected findings in our analysis of kidney disease and duration of residence in the Southeast. First, we found no association of southeastern residence duration with either albuminuria or reduced kidney function, regardless of race. Second, lifetime southeastern residence duration was associated with increased risk of ESRD in blacks only, and we found no evidence of a dose-response association with increasing southeastern residence duration. Finally, blacks (but not whites) with partial southeastern residence duration were possibly less likely to have albuminuria than those who lived their entire lives either within or outside of the Southeast. Residential history data are not commonly collected in cohort studies. However, these unexpected results regarding the effects of geography, and particularly residence in the Southeast, on the prevalence and incidence of kidney disease among blacks and whites may provide a motivation for the collection and examination of more granular geographic data, including both location and mobility, in prospective cohort studies around the world.

## Methods

### Study design and population

The REGARDS study is a national population-based cohort study that recruited 30,239 community-dwelling participants aged **≥**45 years, of whom 55% were women, 42% were black, and 58% were white. Enrollment of the cohort occurred between 2003 and 2007. Approximately 56% of the cohort was recruited from the southeastern states, and the remaining 44% resided in the other 40 contiguous states. Exclusion criteria were race/ethnicity other than non-Hispanic African-American (black) or white, malignancy or other medical conditions that prevented long-term participation in the study, cognitive impairment, nursing home residence, or non-English spoken language. Consent was obtained verbally and later in writing. The study was approved and monitored by institutional review boards at all participating institutions.

### Data collection

Detailed study methods have been published previously [26]. Briefly, individuals were identified from commercially available lists of residents, and recruited using an initial mailing followed by telephone contact. Using a computer-assisted telephone interview, trained interviewers obtained demographic information and medical history. A brief physical exam including blood pressure measurements and collection of blood and urine samples was conducted in-person (usually in the home) 3-4 weeks after the telephone interview. A review of medication pill bottles was conducted as well. Self-administered questionnaires were left with the participant to be completed after the in-person visit and returned by self-addressed prepaid envelopes. Follow-up interviews are attempted every 6 months, and there is a standard protocol for tracking missed contacts.

#### Southeastern residence duration

Duration of residence in the southeastern United States (North Carolina, South Carolina, Georgia, Alabama, Mississippi, Tennessee, Arkansas, and Louisiana) was determined at baseline by a self-administered study residential history questionnaire (“Places You Have Lived Questionnaire”), on which participants report the location (city/state) of all places where they had lived for at least 1 year, from birth to study enrollment, and their ages at each move. Southeastern residence duration was defined as (time lived in the Southeast)/(time lived in the United States)*100. Years lived out of the country, or in unknown locations, were not included (affecting *n* = 4725 participants, median of 5.5 years excluded). Details of the process for matching reported cities with U.S. Census data to minimize misspecification due to misspellings or other mistakes have been published previously [5]. Briefly, participant reports of city/state were matched with U.S. Census Bureau files; in the event of a mismatch, the 10 mnemonically closest city/state combinations were reviewed and evaluated as to the strength of the match by study staff, and those participants with addresses that lacked a strong match at the end of the review process were removed. For analyses, southeastern residence duration was categorized as none (0%), less than half (>0 and <50%), half or more (≥50% and <100%), and all (100%) of the participant’s life, to allow for assessment of dose-response trends and to maintain statistical power without subgroups.

#### Albuminuria

Urine albumin and creatinine were measured using a BN ProSpec Nephelometer (Dade Behring, Marburg, Germany; interassay coefficient of variation, 2.2-4.3%) and Modular-P chemistry analyzer (Roche Hitachi, Indianapolis, IN; interassay coefficient of variation, 2.6-8.6%), respectively [27]. Albuminuria was defined as a baseline urinary albumin:creatine ratio (ACR) ≥ 30 mg/g.

#### Reduced kidney function

Serum creatinine was measured by colorimetric reflectance spectrophotometry using the Ortho Vitros Clinical Chemistry System 950IRC instrument (Johnson & Johnson Clinical Diagnostics, New Brunswick, NJ). eGFR was calculated from isotope dilution mass spectrometry-traceable serum creatinine values using the CKD-EPI equation [28]. Reduced kidney function was defined as a baseline estimated glomerular filtration rate (eGFR) <60 ml/min/1.73 m^2^, corresponding to stage 3-5 (moderate-severe) CKD [29]. Sensitivity analyses using a stricter cutoff of eGFR <45 ml/min/1.73 m^2^ (stages 3B-5), which are more likely to be consistently reduced on multiple measurement, were also conducted.

#### ESRD

The incidence of ESRD was determined by linked data from the United States Renal Data System registry, which records >90% of incident ESRD in the United States. Follow-up time included time from study enrollment to first ESRD treatment, death, or last date of follow-up (9/1/09; mean follow-up, 3.4 years). Participants who initiated ESRD prior to enrollment were excluded from these analyses, and included participants were censored at death or last date of follow-up.

#### Potential confounders

Age, sex, race (white and black), income (<$20,000, $20-34,999, $35,000-74,999, and > $75,000), education (less than high school, high school graduate, some college, college graduate), insurance (yes/no), doctor/clinic for regular medical care (yes/no), self-reported use of oral hypoglycemics/insulin or anti-hypertensive medications (yes/no), current smoking (yes/no), immediate family history of ESRD (yes/no), and use of drugs (yes/no) known to affect CKD and its progression [non-steroidal anti-inflammatory drugs, angiotensin-converting enzyme inhibitors (ACEIs), and angiotensin receptor blockers (ARBs)] were all collected at baseline. Height, weight, and blood pressure were measured during the in-home exam. Blood pressure (average of two measurements) was measured per a standard protocol by a trained technician after the participant rested for 5 minutes, using an appropriately sized cuff and an aneroid sphygmomanometer (American Diagnostic Corporation, Hauppage, NY). Glucose was measured by colorimetric reflectance spectrophotometry using the Ortho Vitros Clinical Chemistry System 950IRC instrument (Johnson & Johnson Clinical Diagnostics, New Brunswick, NJ). Body mass index (BMI) was calculated as weight (kg) divided by height (m) squared. Diabetes was defined as self-reported use of oral hypoglycemics or insulin, fasting glucose **≥**126 mg/dl, or random glucose **≥**200 mg/dl. Hypertension was defined as self-reported use of antihypertensive medication, systolic blood pressure **≥**140 mmHg or diastolic blood pressure **≥**90 mmHg.

### Statistical analyses

Participant characteristics were compared across category of southeastern residence duration using *χ*^2^ or ANOVA tests for categorical and continuous variables, respectively. Crude prevalence of albuminuria and reduced kidney function and crude incidence of ESRD were calculated by residence category and race. Race-stratified logistic models were used to determine the adjusted associations [prevalence odds ratios (PORs)] between southeastern residence duration and the prevalence of albuminuria and reduced kidney function. Race-stratified Cox proportional hazards models of time to ESRD were used to obtain hazard ratios (HRs) of incident ESRD by race and southeastern residence duration. Groups of covariates were entered sequentially to the models to assess confounding: demographics (age, sex), then socioeconomic status (SES; income, education), and finally clinical (diabetes, hypertension). Sensitivity analyses were performed in which reduced kidney function was defined by eGFR <45 ml/min/1.73 m^2^. Further sensitivity analyses using evenly spaced (at 20% increments, excluding 0% and 100%) cutoffs and using a dichotomous variable assessing early-life southeastern residence (first 18 years spent in the Southeast, yes vs. no) were also performed. Finally, potential effect modification by income (with higher income potentially protecting individuals from any negative regional environmental effect) was assessed via examination of the association within low- and high-income groups (<$35,000 and > $35,000 annual income, respectively). Potential effect modification by age, which might indicate a cohort effect, wherein effects may be weaker in those born later (due to society-level changes over time), was also assessed. The statistical significance of interaction terms was assessed with Wald *χ*^2^ tests. For this study, participants were limited to those with lifetime residence information (*N* = 27,070); of these participants, those with complete information for assessment of prevalent albuminuria (*N* = 25,797) and reduced kidney function (*N* = 25,965) and free of ESRD at the start of the study (*N* = 26,950) were included in respective analyses. All analyses were performed using SAS v. 9.2 (SAS Institute, Cary, NC) and Stata v. 12 (StataCorp, College Station, TX).

## Competing interests

The authors declare that they have no competing interests.

## Authors’ contributions

LP carried out the analysis and draft the manuscript; VJH participated in the design and coordination of the study and provided feedback on the written manuscript, SJ, PM, DR, DTL, and DGW provided feedback on the analyses and drafts of the manuscript; GH served as principal investigator of the REGARDS study and provided feedback on drafts of the manuscript; and WMM served as primary mentor to LP and helped to design and perform the analyses and draft the manuscript. All authors read and approved the final manuscript.
